# Hemolysin coregulated protein 1 as a molecular gluing unit for the assembly of nanoparticle hybrid structures

**DOI:** 10.3762/bjnano.7.32

**Published:** 2016-03-04

**Authors:** Tuan Anh Pham, Andreas Schreiber, Elena V Sturm (née Rosseeva), Stefan Schiller, Helmut Cölfen

**Affiliations:** 1 Department of Chemistry, Physical Chemistry, University of Konstanz, Universitätstrasse 10, D-78457 Konstanz, Germany,; 2 Zentrum für Biosystemanalyse (ZBSA), Albert-Ludwigs-Universität Freiburg, Habsburgerstrasse 49, D-79104 Freiburg, Germany

**Keywords:** gold catalyst, hemolysin coregulated protein 1 (Hcp1), magnetic hybrid materials, nanoparticles, self-assembly, SERS

## Abstract

Hybrid nanoparticle (NP) structures containing organic building units such as polymers, peptides, DNA and proteins have great potential in biosensor and electronic applications. The nearly free modification of the polymer chain, the variation of the protein and DNA sequence and the implementation of functional moieties provide a great platform to create inorganic structures of different morphology, resulting in different optical and magnetic properties. Nevertheless, the design and modification of a protein structure with functional groups or sequences for the assembly of biohybrid materials is not trivial. This is mainly due to the sensitivity of its secondary, tertiary and quaternary structure to the changes in the interaction (e.g., hydrophobic, hydrophilic, electrostatic, chemical groups) between the protein subunits and the inorganic material. Here, we use hemolysin coregulated protein 1 (Hcp1) from *Pseudomonas aeruginosa* as a building and gluing unit for the formation of biohybrid structures by implementing cysteine anchoring points at defined positions on the protein rim (Hcp1_cys3). We successfully apply the Hcp1_cys3 gluing unit for the assembly of often linear, hybrid structures of plasmonic gold (Au NP), magnetite (Fe_3_O_4_ NP), and cobalt ferrite nanoparticles (CoFe_2_O_4_ NP). Furthermore, the assembly of Au NPs into linear structures using Hcp1_cys3 is investigated by UV–vis spectroscopy, TEM and cryo-TEM. One key parameter for the formation of Au NP assembly is the specific ionic strength in the mixture. The resulting network-like structure of Au NPs is characterized by Raman spectroscopy, showing surface-enhanced Raman scattering (SERS) by a factor of 8·10^4^ and a stable secondary structure of the Hcp1_cys3 unit. In order to prove the catalytic performance of the gold hybrid structures, they are used as a catalyst in the reduction reaction of 4-nitrophenol showing similar catalytic activity as the pure Au NPs. To further extend the functionality of the Hcp1_cys3 gluing unit, Fe_3_O_4_ and CoFe_2_O_4_ NPs are aligned in a magnetic field and connected by utilization of cysteine-modified Hcp1. After lyophilization, a fiber-like material of micrometer scale length can be observed. The Fe_3_O_4_ Hcp1_cys3 fibers show superparamagnetic behavior with a decreasing blocking temperature and an increasing remanent magnetization leading to a higher squareness value of the hysteresis curve. Thus the Hcp1_cys3 unit is shown to be very versatile in the formation of new biohybrid materials with enhanced magnetic, catalytic and optical properties.

## Introduction

**Figure 1:**
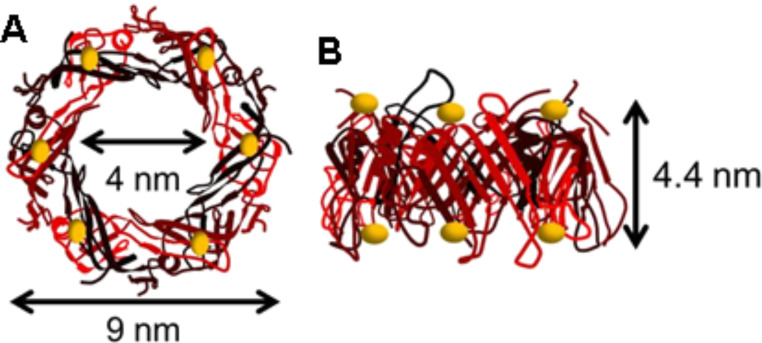
A) In the top-view the toroidal, the homohexameric structure of the Hcp1_cys3 mutant of Hcp1 shows a cavity with a diameter of 4 nm and an outer diameter of 9 nm. On the top of the protein ring, six cysteine groups (yellow dots) provide the binding sites for the NPs. B) The side-view the Hcp1_cys3 structure shows a height of 4.4 nm and the cysteine groups (yellow dots) on the top and bottom (PDB code: 1Y12).

Self-assembly plays a pivotal role in bottom-up strategies for the synthesis of advanced nanostructures [1]. The resulting assemblies can be one-, two- or three-dimensional. One-dimensional nanostructures show particularly great promise due to their large anisotropy in shape and possible properties. However, nanoparticle (NP) assembly leading to one-dimensional (1D) superstructures or arrays has received less attention compared to their two- or three-dimensional equivalents [2]. The NP assembly can be conducted in a template-based or template-free way. In particular, a template-free approach is more difficult to achieve since specific interactions in terms of chemical and spatial interplay have to be ensured. The controlled assembly of NPs using organic compounds such as polymers [3–7], peptides [8–9] and DNA [10–14] demonstrate great potential in the design of 1D NP hybrid structures with advanced properties. However, examples for NP assembly using proteins are still limited [15–18]. Here, we apply the hemolysin coregulated protein 1 (Hcp1) homohexameric protein from *Pseudomonas aeruginosa* with its toroidal structure as a nanotechnological building block [19–20] (protein data bank (PDB) code: 1Y12) for the fabrication of magnetically and plasmonically active assemblies. The cysteine-modified mutant (Hcp1_cys3) of the native Hcp1 protein is proven to be a great candidate, triggering the assembly of CdSe quantum dots and Au NPs into 1D chains and network structures [21–22]. Due to the genetic modification of the native protein structure with cysteine on the top and bottom of the ring, the resulting Hcp1_cys3 mutant provides specific binding sites for different metallic NPs (Figure 1). Through these defined interaction points, the protein is able to connect NPs in the same size range of the protein to chain structures in a “Lego-like” manner. Utilizing Hcp1_cys3 in this work, we extend the protein-adaptor-based nano-object assembly (PABNOA) approach to guide the formation of magnetic NPs as a new class of inorganic nanomaterials. Furthermore, kinetic investigation of the formation of such 1D Au NP structures and the utilization of this structure, for example, as a SERS template and catalyst are also of great interest. The formation kinetics of Au NP networks triggered by Hcp1_cys3 is investigated using UV–vis spectroscopy, TEM and cryo-TEM. Since the Hcp1_cys3 protein in the Au NP assembly is located at the interstitial sites of the Au NPs, strong Raman signal enhancement of Au NP chains formed with Hcp1_cys3 can be observed. The resulting Raman spectrum indicates a stable secondary structure of Hcp1 and the Hcp1–Au NP connection via gold–thiol binding. The Au NP network shows similar reactivity to the colloidal Au NPs as a catalyst in the reduction reaction of 4-nitrophenol to 4-aminophenol. To explore the broad application of our concept, Hcp1_cys3 is also applied to assemble Fe_3_O_4_ and CoFe_2_O_4_ NPs. The reaction is conducted under an external magnetic field. After lyophilization of the reaction mixture, fiber-like structures in the micrometer range are obtained. The TEM investigation demonstrates networked structures of Fe_3_O_4_ and CoFe_2_O_4_ NPs. The magnetic measurements reveal a superparamagnetic character for the Fe_3_O_4_ Hcp1_cys3 material with decreasing blocking temperature.

## Results and Discussion

### Gold nanoparticle assembly

**Figure 2:**
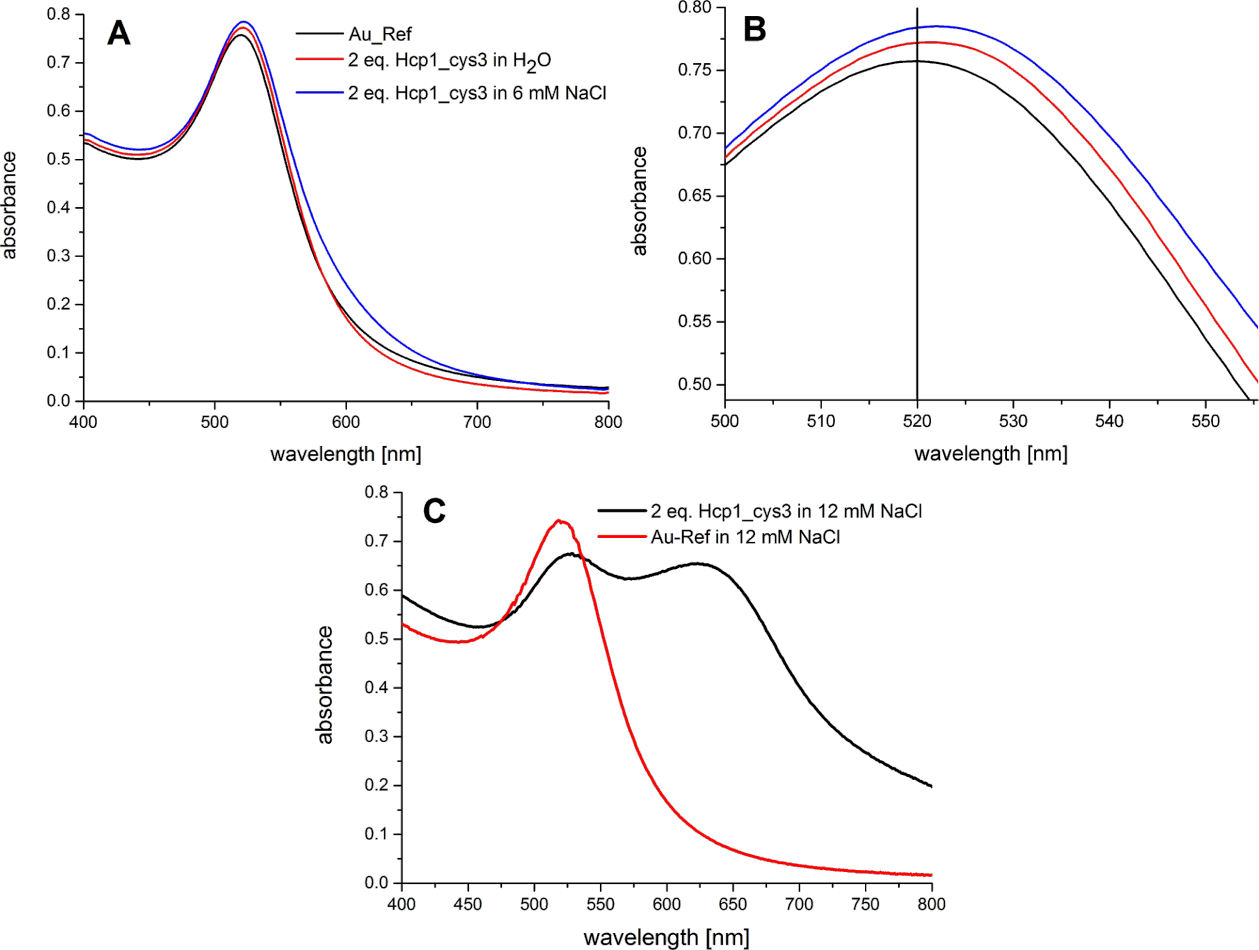
A) UV–vis spectra of Au NPs containing 2 equiv Hcp1_cys3 with 0 and 6 mM NaCl concentration. B) Details of the UV–vis spectra in A). C) UV–vis spectra of Au NP mixture containing 12 mM NaCl with and without 2 equiv Hcp1_cys3.

The Au NPs used in the following experiments have a mean diameter of 10.7 ± 2.0 nm as obtained from TEM (Figure S1, Supporting Information File 1). All samples were prepared using the same protocol as described in the Experimental section. First, we investigated the influence of ionic strength on the assembly process of Au NPs. In Figure 2, the UV–vis spectra of a Au NP solution with 2 equiv Hcp1_cys3 at different ionic strengths are shown. At low ionic strength (0–6 mmol/L) the surface plasmon resonance peak of Au NP at 520 nm shifts very slightly to 522 nm (Figure 2A,B), which indicates the protein binding to Au NP [23]. This observation is consistent with previously published results [22] which report on M2F03-antibody-functionalized Au NPs. In solution, these exhibit an increasing antigen concentration with a maximal shift of 3 nm of the SPR peak [23]. As the ionic strength is increased to 12 mM, two peaks at 520 nm and 645 nm can be observed (Figure 2C). The first peak is related to the transversal resonance and the second peak to the longitudinal resonance, predominantly observed in linear gold nanostructures such as nanorods [24] and chains of gold spheres [25]. It is remarkable that the longitudinal resonance peak is only observed for the Au NP sample with 2 equiv Hcp1_cys3 and not for the citrate-stabilized initial Au NPs. This shows that the formation of the larger linear assemblies only takes place for the Hcp1_cys3-functionalized NPs, where the citrate-stabilized Au NPs remain stable as evidenced by their unchanged surface plasmon resonance at 520 nm. When the NaCl concentration exceeded 12 mM, a prompt color change to blue followed by precipitation of a blue solid was observed (data not shown here). Hence, 12 mM seemed to be the ideal ionic strength to trigger the assembly of Au NPs.

**Figure 3:**
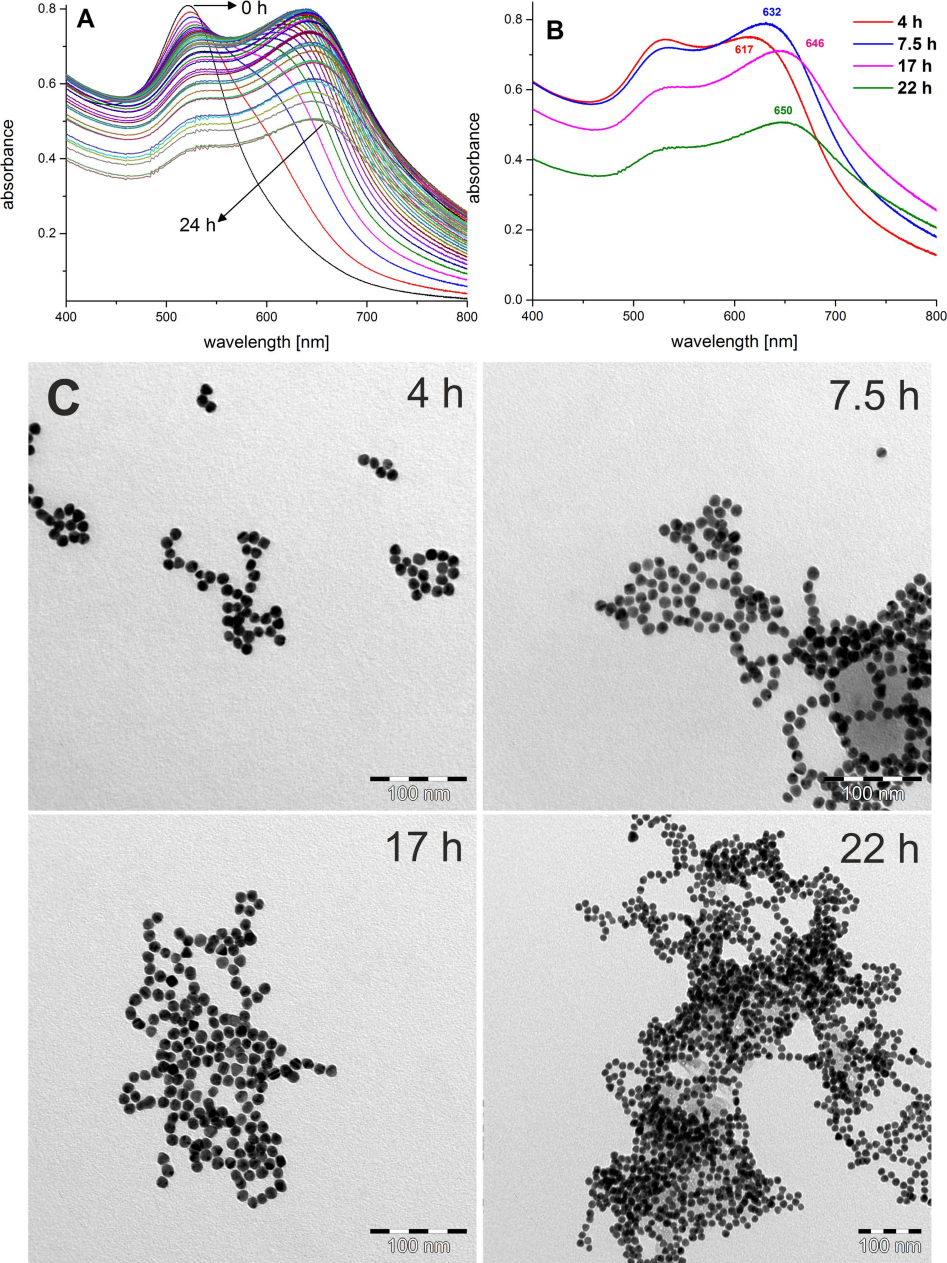
A) Overview of time-resolved UV–vis spectra during the self-assembly reaction of the Hcp1_cys3-functionalized Au NPs at 12 mM ionic strength over 24 h. Every 30 min a UV–vis spectrum was taken. B,C) Single UV–vis spectra at different times and the corresponding TEM images indicate the assembly Au NPs of different morphology, from short chains to an open network.

For the kinetic investigation of the Au NP assembly, UV–vis spectra were recorded for 24 h at a time interval of 30 min. At the same time, samples were also taken for TEM measurements. In the overview spectra (Figure 3A) a red shift of the transversal 520 nm plasmon resonance peak to 530 nm is observed with time and the appearance of a second longitudinal peak first around 617 nm then shifting to 650 nm at the end of the reaction. This peak position around 650 nm stays constant after 18 h. In Figure 3B, UV–vis spectra with the most distinguished optical change at different times are shown. The TEM images of the corresponding spectra in Figure 3C indicate the formation of a Au network starting with the formation of short and long chains containing 3–10 Au NPs. These chains form a network structure of increasing size as the reaction proceeds. In comparison to the TEM images, the UV–vis spectra clearly indicate the formation of linear architectures of Au NPs. This is shown in Figure 3B where the UV–vis spectra for four samples are shown, which were investigated at different experimental times. The shift of the longitudinal plasmon resonance peak with time is obvious, indicating the increasing length of linear Au NP chain structures. In addition, the ratio between the longitudinal and transversal surface plasmon resonance peaks becomes larger with time, further indicating the increasing elongation of the NPs assembly structures. The decrease in the absorbance between 17 h and 22 h is due to the slight precipitation of large Au Hcp1_cys3 structures.

**Figure 4:**
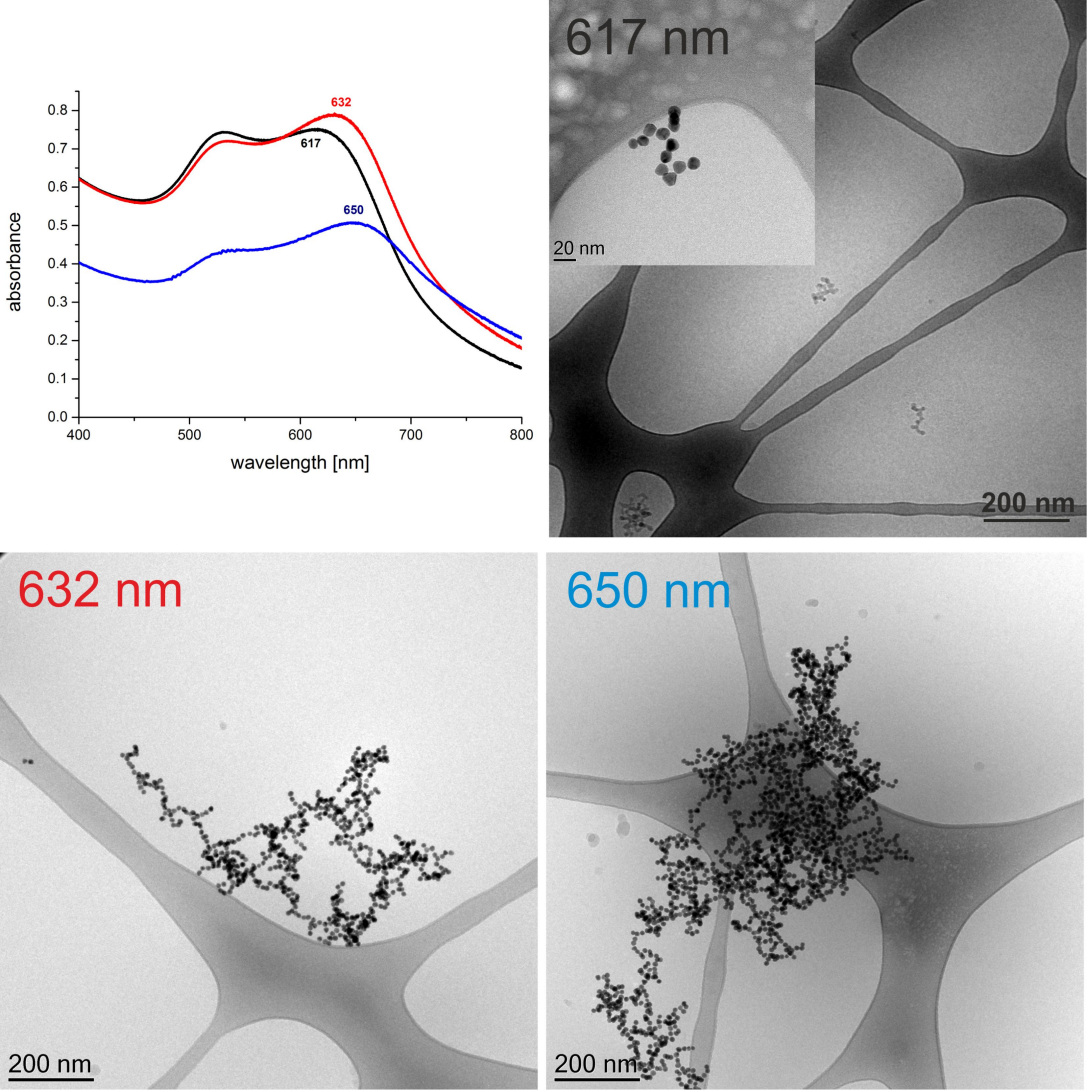
Cryo-TEM results by taking samples during the self-assembly reaction of the Hcp1_cys3-functionalized Au NPs at 12 mM ionic strength. The observed architectures are related to the UV–vis spectra.

For better visualization of the different Au NP architectures, a cryo-TEM investigation was conducted for 3 samples at 7.5 h, 17 h and 22 h, as shown in Figure 4. The formation kinetics observed by UV–vis and cryo-TEM correspond very well, showing short chains of Au NPs with a second peak around 617 nm. As the longitudinal peak shifts to 632 nm, longer chains of 500 nm length with the branching behavior can be observed. At the end, an open network of Au NPs on the microscale exhibits a broad second peak at 650 nm.

**Figure 5:**
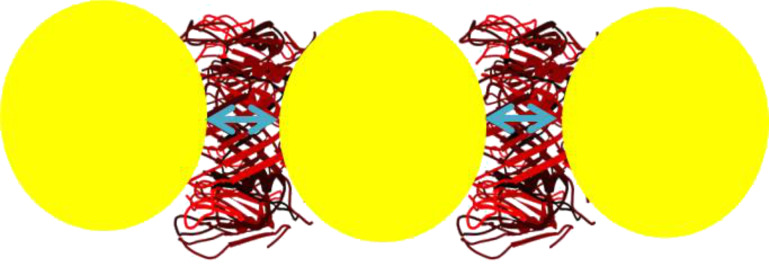
The interparticle distance of Au NPs was determined by calculation and by measuring the distances between the Au NPs in the network structure in a TEM image, resulting in theoretical and experimental values of 3.62 nm and 2.8 ± 0.6 nm, respectively.

By measuring the distance between the Au NPs in the TEM image, as illustrated in Figure 5, an interparticle distance of 2.8 ± 0.6 nm was found (Figure S2, Supporting Information File 1), which in the first approximation fits to the calculated distance of 3.62 nm (Equation S1, Supporting Information File 1). The discrepancy between the measured and theoretical values originate from a) the drying effect during sample preparation, which can cause shrinking of the organic material (protein) and b) the nonideal spherical shape of our Au NPs, which allows the penetration of the NP into the protein cavity, leading to a decrease of the interparticle distance.

**Figure 6:**
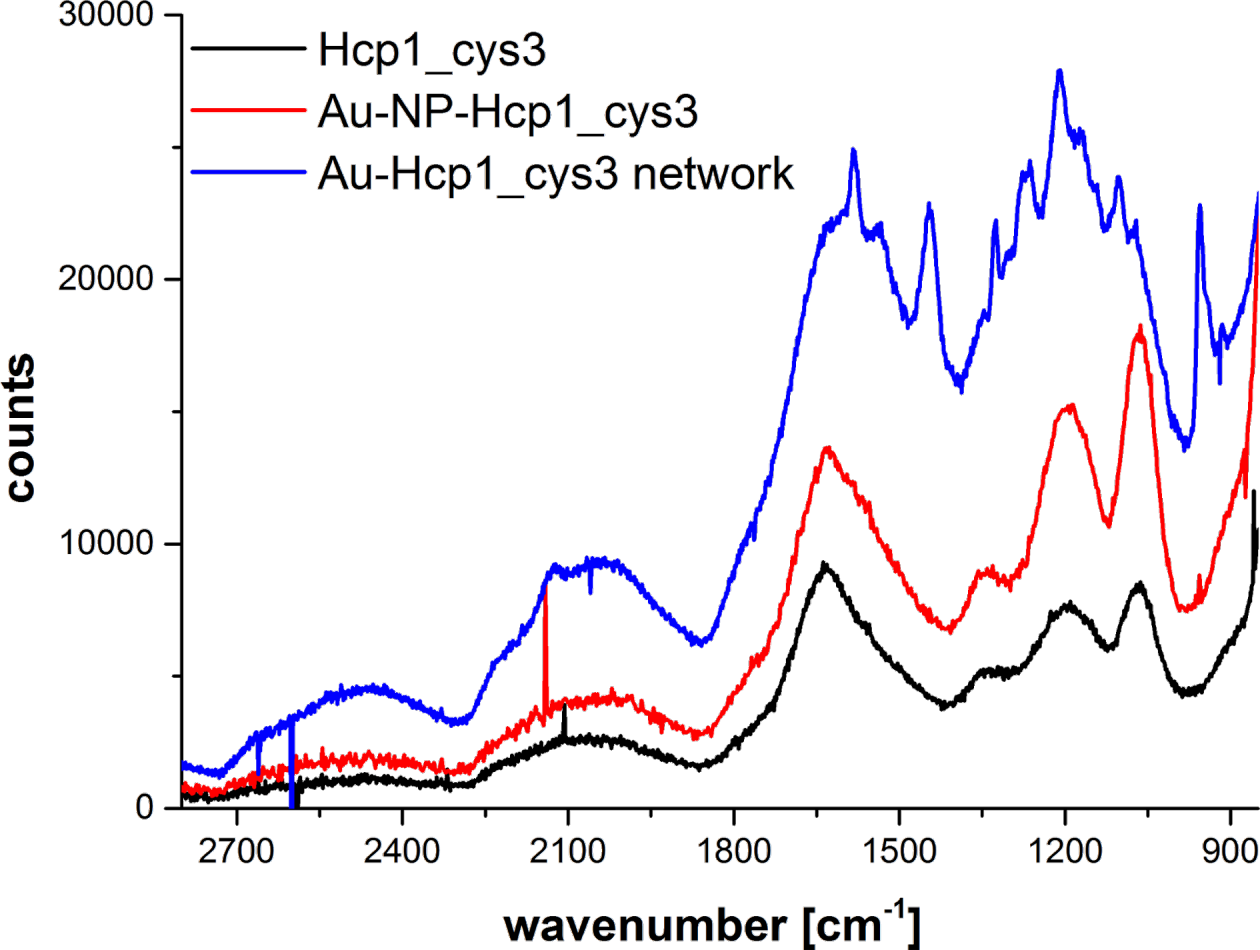
Raman spectra of pure Hcp1_cys3, Au NPs with 2 equiv Hcp1_cys3 in 6 mM NaCl and Au Hcp1_cys3 network. The Raman signal of Hcp1 increased since the protein is located in the hot spot of the Au NP network.

As the TEM distance measurements only indirectly support the assumption that Hcp1_cys3 is located between the Au NPs in this network architecture, further evidence must be obtained. Therefore, Raman spectroscopy was performed. If Hcp1_cys3 is located in between two adjacent Au NPs, as indicated by our TEM investigations, surface-enhanced Raman scattering (SERS) should be observed due to the amplification of the electromagnetic field in this so-called “hot spot” [26]. In Figure 6, the Raman spectra of the pure Hcp1_cys3 Au NP sample with 2 equiv Hcp1_cys3 in 6 mM NaCl from Figure 2A and the Au Hcp1_cys3 network from Figure 3 and Figure 4 are shown. These samples represent 3 cases: a) pure/bare Hcp1_cys3 protein, b) Hcp1_cys3 adsorbed on the Au NP surface and c) Hcp1_cys3 located in the hot spot. The Raman intensity accompanied by the resolution increases from the pure protein to protein adsorbed on Au NP and reaches its maximum in the networked sample. The enhancement by factors of 8·10^4^ for the bands at 1210 and 1590 cm^−1^ is significant and proves the prediction of signal enhancement in the Au NP hot spot and the interstitial position of Hcp1_cys3 between two Au NPs in the Au NP network. The magnitude of the signal enhancement observed here is similar to the enhancement of 10^5^ reported for BSA protein on gold nanocylinders [27]. The identification of the Raman bands in the Au NP network spectra reveals further interesting information. The typical amide I, II, III bands for protein can be identified. The Raman band at 1590 cm^−1^ of the C=O stretching mode can be assigned the amide I band for a β-sheet structure [28]. The amide II, related to N–H bending (which is normally very weak in Raman spectra), occurs due to resonant excitation of Au NPs at 1535 cm^−1^. This position of the amide II band is typical for parallel β-sheet structures [28]. The amide III related to N–H bending and C–N stretching is split in two bands at 1281 cm^−1^ and 1210 cm^−1^, which is related to α-helix and β-sheet structures [29]. Here, the β-sheet band also has a higher intensity than the α-helix band. Since each monomeric unit of the Hcp hexamer contains 10 β-sheet and 1 α-helix structures [19], the dominance of the β-sheet structure in the amide I, II and III bands exhibits a stable secondary structure of the Hcp protein on the Au NP surface. This further demonstrates the stability of the entire protein structure after binding to the Au NP, since it is well known that the adsorption of proteins on a NP surface can disturb this structure [30]. Furthermore, the C–COO^−^ and C–H stretching bands of cysteine appear at 955 cm^−1^ [31–32] and 1105 cm^−1^ [33]. The strong S–H band of cysteine around 2600 cm^−1^ is missing due to the direct binding of the sulfur atom to the NP surface [32]. Based on the Raman investigation, it can be concluded that Hcp1_cys3 is located in between two Au NPs, leading to a signal enhancement of the protein. The secondary protein structure remains intact on the NPs surface and the protein–Au binding takes place via the sulfur in the integrated cysteine group.

**Figure 7:**
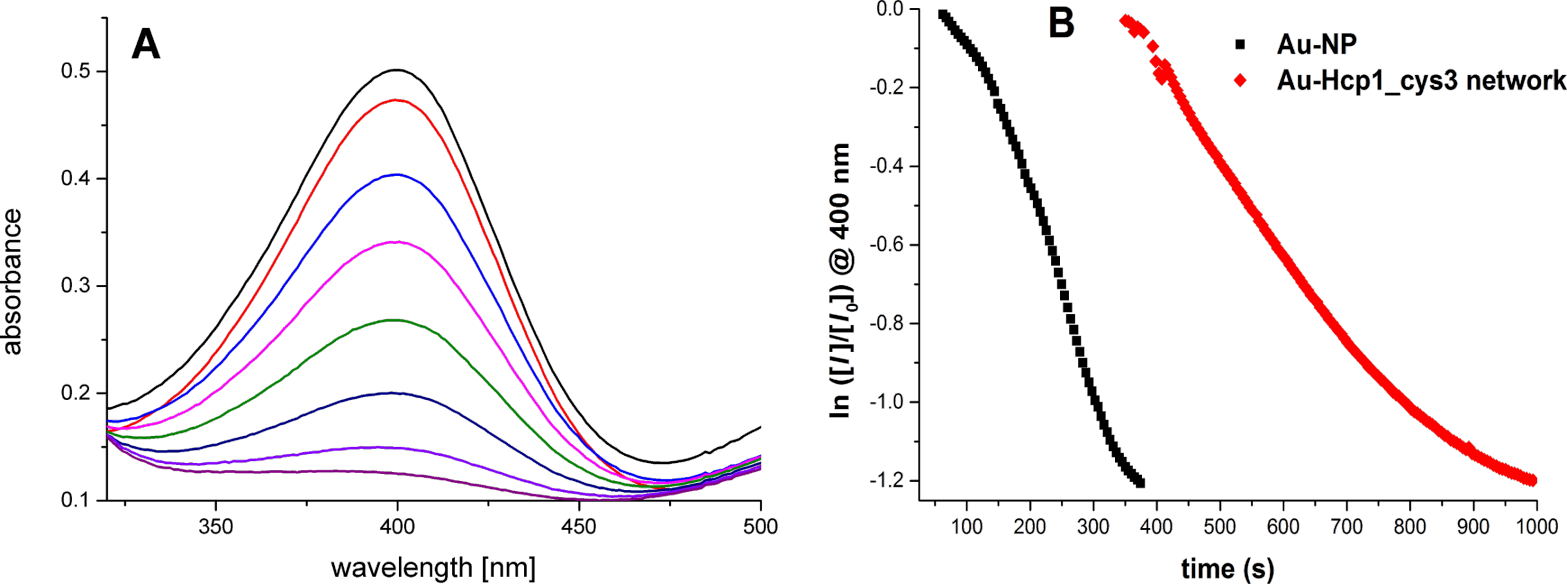
A) UV–vis spectra of a 4-nitrophenol solution at different reaction times after the addition of the catalyst showing decreased absorbance. B) The time-dependent absorbance change at 400 nm in a mixture with pure Au NPs and the Au network as catalyst.

In order to demonstrate a first application of such protein-based hybrid structures, we use our Au NP network as a catalyst in the reduction reaction of 4-nitrophenol. The reduction of 4-nitrophenol to 4-aminophenol is a standard reaction to evaluate the catalytic reactivity of NPs [34]. The evaluation is based on the time-dependent absorbance decrease of the 4-nitrophenol cation at 400 nm. The absorbance change at 400 nm (ln *I*/*I*_0_ at 400 nm, where *I*_0_ is the absorbance at *t* = 0 s) was linearly fitted to obtain the reactivity constant, *k*. In Figure 7 the UV–vis spectra of the 4-nitrophenol solution during the catalytic reaction and the absorbance change at 400 nm are shown. The Au network shows a smaller *k* value of (2.23 ± 0.51) × 10^−3^ s^−1^ than the Au NPs of (3.24 ± 0.81) × 10^−3^ s^−1^. This could be due to the occupation of Au NP surface by the proteins, leading to a decreased reactive surface and reduction rate. Nevertheless, this *k* value is in the range of Au NPs of similar size [35]. Taking advantage of the larger size, this catalyst can be easily recycled by centrifugation or even by filtration compared to the pure Au NPs. This makes the hybrid structure more attractive as a catalyst with comparable reactivity.

### Magnetic nanoparticle assembly

**Figure 8:**
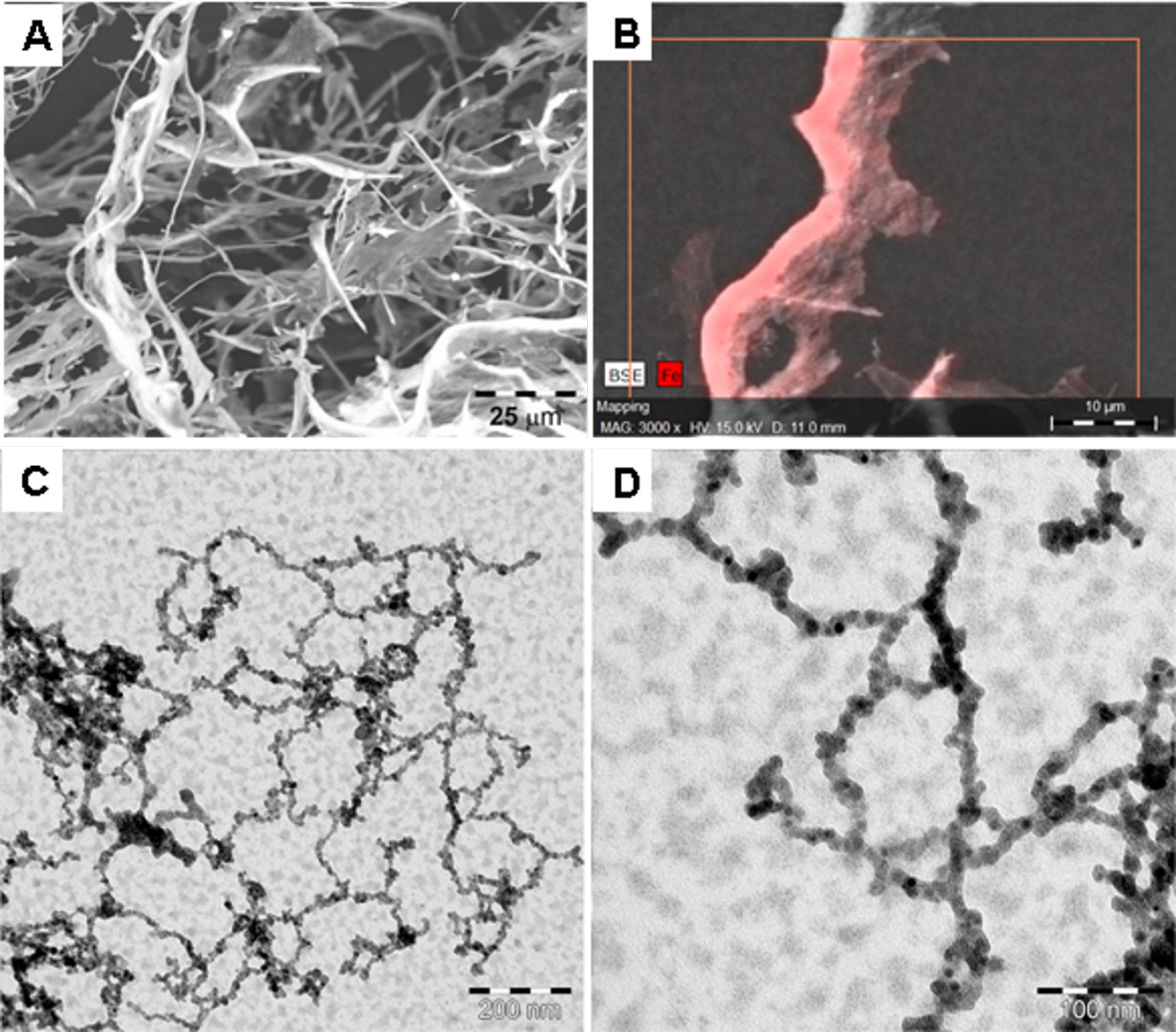
A) and B) SEM image and EDX analysis of Fe_3_O_4_ Hcp1_cys3 fiber-like structure after lyophilization. C) and D) TEM images show a network of magnetite NPs, which are arranged in linear chains.

In this work, the concept of NP network formation using Hcp1_cys3 as a connecting unit is extended to magnetite NPs (Fe_3_O_4_ NPs) and cobalt ferrite NPs (CoFe_2_O_4_ NPs). The synthesis of magnetite NPs with a size of about 8 nm and the ligand exchange followed the protocol of Cabrera et al. [36], with results as shown in the TEM images of Figure S4, Supporting Information File 1. The water-dispersible, mercaptosuccinic acid stabilized Fe_3_O_4_ NP solution was aligned in an external magnetic field parallel to the sample. After the protein addition, Hcp1_cys3 interconnected the NPs, which results in a fibrous bio-hybrid structure. In Figure 8, SEM with EDX analysis and the TEM images of the resulting hybrid material are shown. Fiber-like structures with lengths of 30–100 μm and widths of 1–0.5 μm can be observed (Figure 8A). The EDX analysis reveals the homogenous distribution of the Fe content in these fibers (Figure 8B). The reference sample (with only Fe_3_O_4_ NPs) prepared by the same protocol show spherical aggregates of 10–20 μm in diameter (data not shown here). A control sample was prepared by mixing Fe_3_O_4_ NP and Hcp1_cys3 solutions at 12 mM ionic strength, similar to the Au network sample. In this case, NP aggregation without any orientation can be observed in TEM images (Figure S5, Supporting Information File 1). These results indicate that the prealignment of Fe_3_O_4_ NPs in an external magnetic field is essential for the linear arrangement of the NPs chain formation. On the other hand, TEM investigations of the hybrid material show a network of magnetite NPs arranged in linear chains (Figure 8C,D). In contrast to the Au NPs, which give a higher TEM contrast, no clear NP separation could be observed between the individual NPs. However, the interparticle distance of the Fe_3_O_4_ NPs in the high resolution TEM (HRTEM) image in Figure S6, Supporting Information File 1 and by calculation following Equation S1, Supporting Information File 1, reveals a value of 1.5 ± 0.5 nm and 3.32 nm. The reason for the large discrepancy compared to the calculated value can be caused by the smaller size of the Fe_3_O_4_ NPs, leading to higher penetration depth into the protein cavity. Additionally, artefacts, such as drying effects during the TEM sample preparation, can also decrease the interparticle distance.

**Figure 9:**
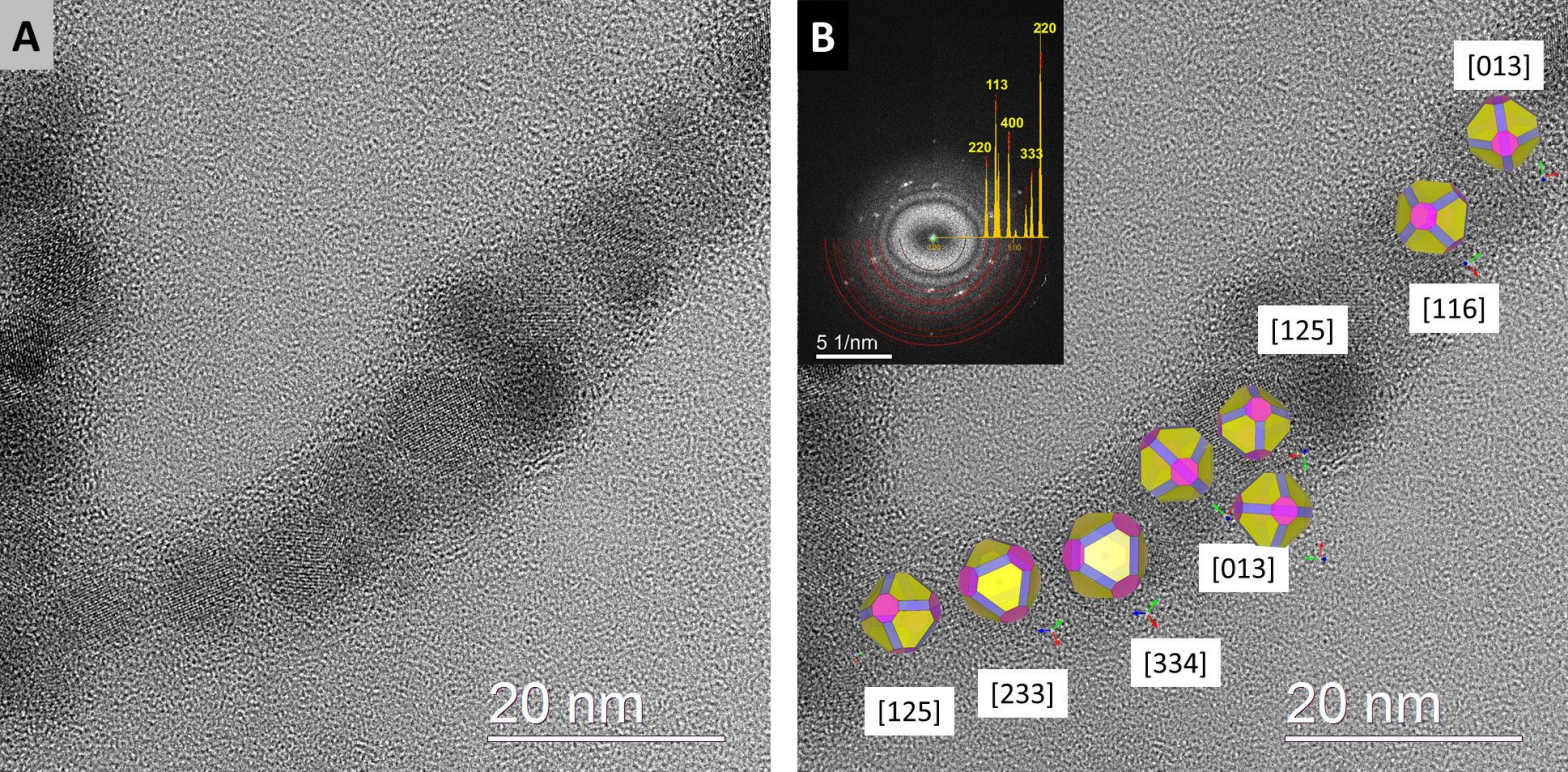
A) Overview HRTEM image of magnetite NPs in the Fe_3_O_4_ Hcp1_cys3 fiber. B) HRTEM image from (A) overlaid with the orientation map of magnetite nanocrystals derived from the analyses of the FFTs of HRTEM images of individual particles. The FFT retrieved from the whole chain is shown in the inset and is overlaid with the diffraction pattern of magnetite.

In order to evaluate the orientational relationship between the iron oxide NPs within the assembled fiber-like structure, HRTEM images were recorded with a large field of view. Figure 9A illustrates one of the HRTEM images (“snapshot”) which was used to determine the orientation of magnetite along the 1D chain by evaluation of fast Fourier transforms (FFT) of individual NPs. Since the synthetic magnetite NPs are quite inhomogeneous in morphology, for illustrational purposes (to visualize the orientation along the chain), the shape of the NPs was approximated as rhombicuboctahedra. This is a common shape for this crystalline material (the sets of <100>, <111> and <110> facets are colored in pink, yellow and purple, respectively). The FFT retrieved from the whole chain (inset in Figure 9B) illustrates the preferable orientation of NPs (characterized by spot and arc-like reflections at the diffractogram). In Figure 9B the orientation map of magnetite NPs derived from the high-resolution micrograph is displayed. The FFT analysis revealed that the particles are viewed from [233], [334], [013], [125], [116] zone axes. In this case, the orientational mismatch between the neighboring NPs (along the viewing direction) vary from 9° to 35°. Furthermore, these orientations are very close to the main zone axis [111] (in case of [233], [334] the orientational mismatches are 10° and 8°) and [001] (in case of [013], [125], [116], the orientational mismatches vary from 13° to 24°). This indicates that during the aggregation and lyophilization processes, magnetite NPs (stabilized by protein molecules) have a tendency to adjust to a preferable crystallographic orientation perpendicular to the fiber elongation.

**Figure 10:**
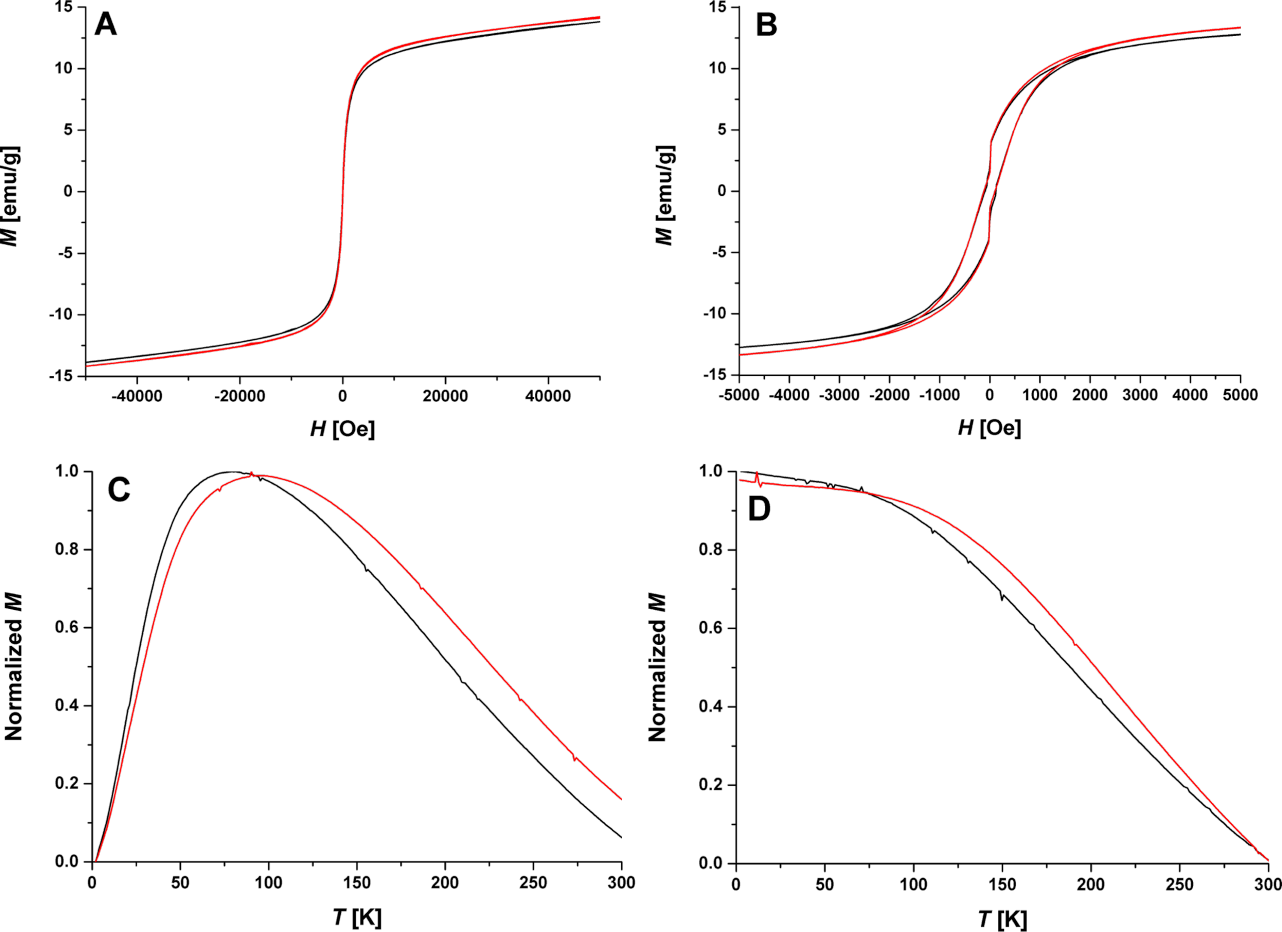
A) and B) Hysteresis curves at 300 K and 2 K, respectively. C) ZFC curves of the fibers and pure NPs, which show a decrease of *T*_b_. D) FC curves indicate an increasing slope. Black line: Fe_3_O_4_ Hcp1_cys3 fibers, red line: Fe_3_O_4_ NPs as reference.

Finally, magnetic measurements of the hybrid material were conducted. In Figure 10A, results of a superconducting quantum interference device (SQUID) measurement show that the hybrid material is superparamagnetic at room temperature with saturation magnetization (*M*_S_) of 12.81 emu/g, which is similar to the blank NPs of 13.21 emu/g. The hybrid material demonstrates at 2 K a similar hysteresis curve to the Fe_3_O_4_ NP with *M*_S_ of 13.69 emu/g and remanent magnetization (*M*_R_) of 2.64 emu/g, but with a higher squareness value (*M*_R_/*M*_S_ ratio) of 0.2 (Figure 10B). The Fe_3_O_4_ NPs have a squareness value of 0.15. Since a theoretical squareness value for a uniaxial system of magnetite bulk material is 0.5, our values are in general smaller due to the frustration of the magnetic moment at the NP surface [37]. But obviously the NP assembly can reduce this frustration leading to a higher *M*_R_/*M*_S_ ratio. The coercive field (*H*_C_) also stays unchanged for both samples at 100 Oe. The blocking temperature, *T*_b_, (the maximum in the zero-field cooling (ZFC) curve), decreases from 94 K to 78 K as the hybrid structure is formed (Figure 10C). Hiroi et al. found that decreasing *T*_b_ for γ-Fe_2_O_3_ NPs cores with increasing SiO_2_ shell thickness leads to increasing interparticle distance [38]. In our case, the increase of interparticle distance supports the theory that the Hcp1_cys3 is located between the NPs, as is visible in the HRTEM image (Figure S6, Supporting Information File 1 and Figure 5). The field-cooling (FC) curves exhibit a slight steeper slope in the hybrid material (Figure 10D). However, the overall trend in the FC curves for both samples is similar. The effective magnetic anisotropy constant (*K*_eff_) can be calculated using following expression: *K*_eff_ = (25·*k*_B_·*T*_b_)/*V* [39], where *k*_B_ is the Boltzmann constant and *V* the volume of the NPs. The obtained value is 8.59·10^5^ erg/cm^3^ for Fe_3_O_4_ NP and 7.05·10^5^ erg/cm^3^ for Fe_3_O_4_ NP Hcp1_cys3 fibers. These values are considerably larger than the reported value for Fe_3_O_4_ bulk material (1·10^5^ erg/cm^3^) [39]. The higher value is due to the broken symmetry at the surface or interface of the NPs, which can enhance the surface anisotropy and cause the increased effective values of *K* [40]. The fiber-like hybrid material shows *K*_eff_ close to the bulk material value, which indicates the stabilization of the surface spin compared to the blank Fe_3_O_4_ NPs.

**Figure 11:**
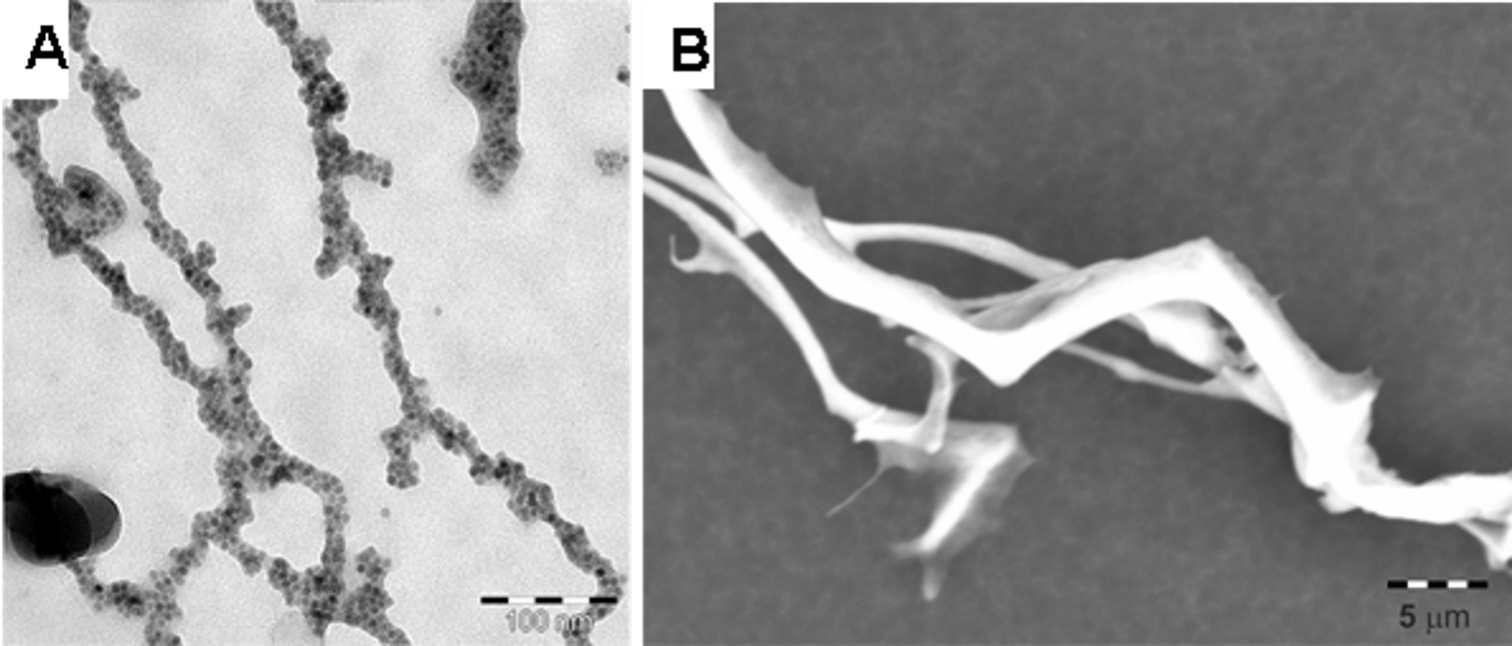
A) TEM and B) SEM images of CoFe_2_O_4_ Hcp1_cys3 sample after lyophilization. Chains of NPs and fiber-like structures on the micrometer length scale for the hybrid fibers can be seen.

A third NP system demonstrates that our concept of the utilization of Hcp1_cys3 as a gluing unit between the NPs to fabricate a linear structure is universal. We synthesized cobalt ferrite NPs (CoFe_2_O_4_ NPs) following the protocol of Cabrebra [36] and exchanged the oleic acid ligand to phosphonoacetic acid by the protocol of Lees [41] to disperse the NPs in water. The phosphonoacetic acid stabilized CoFe_2_O_4_ NPs have a mean diameter of 5.5 nm as determined by TEM (Figure S7, Supporting Information File 1). Following the same protocol as described for the Fe_3_O_4_ Hcp1_cys3 sample preparation, we prealigned the CoFe_2_O_4_ NPs in a magnetic field and added Hcp1_cys3 as a gluing unit to the mixture. After the lyophilization, a linear arrangement of the NPs into chains and fiber-like structures on the microscale scale in length were observed in the TEM and SEM images shown in Figure 11A,B. Similar to the Fe_3_O_4_ NP assemblies, an interparticle distance of 0.8 ± 0.3 nm was determined from the HRTEM image (Figure S8, Supporting Information File 1), which is less than the calculated distance value of 2.67 nm. This result indicates, as already assumed for the Fe_3_O_4_ Hcp1_cys3 structure, a quite high indulgence of the Hcp1 ring structure. This results in a greater penetration depth of the NP into the protein cavity, leading to a smaller interparticle distance.

## Conclusion

In this study, we used the toroidal protein Hcp1_cys3 as a protein adaptor to glue NPs in a “Lego-like” manner into linear chains, resulting in three protein-based NP hybrid structures on the meso- and micro-scale. The Au NP Hcp1_cys3 assemblies show the formation of short chains in the nanometer range which grow to longer branched chains in UV–vis spectroscopy and TEM/cryo-TEM kinetic investigations. Finally, a network structure of Au NPs is formed on the mesoscale. The branches are evident since it can be assumed that the Hcp1 ring statistically adsorbs on Au NPs and even when an equivalent number of protein molecules and NPs are used. A statistical adsorption of two proteins on one NP is possible, leading to branched networks. As a control experiment, an Au NP sample with only 0.6 equiv Hcp1_cys3 was prepared and shows short chains with 2–10 Au NPs and also free Au NPs (Figure S9, Supporting Information File 1). Similar statistical protein adsorption on one NP is also observed for the Fe_3_O_4_ Hcp1_cys3 fiber, leading to the formation a protein layer on the side of the Fe_3_O_4_ NPs in the nanoparticle chains (Figure S10, Supporting Information File 1). By means of the location of Hcp1_cys3 in the Au NP architecture, we revealed a stable secondary protein structure on the Au NP surface and confirmed the binding site of protein to Au on the sulfur atom of the cysteine. Furthermore, the Au network shows similar catalytic reactivity as the blank Au NP, which makes it more attractive owing to the easier recycling condition of the catalyst. This protein gluing unit approach was then extended to assemble Fe_3_O_4_ NPs and CoFe_2_O_4_ NPs. The network structure of NPs on the mesoscale and fiber-like hybrid material on the micrometer scale were synthesized by prealignment of NPs in an external magnetic field and utilization of added Hcp1 as the connecting unit. The HRTEM investigation shows a preferable crystallographic orientation of magnetite NPs in the fiber-like structure. The results of the magnetic measurements exhibit similar *M*_R_ and *M*_S_ values at room temperature and low temperature for the hybrid material and NPs. The hybrid materials reveal a lower blocking temperature than the blank NPs due to protein incorporation between the NPs, leading to an enhancement of the magnetic spin in system.

In conclusion, we could utilize the cysteine-modified toroid protein Hcp1_cys3 as an effective glue to form linear chains of NPs to extend the previously reported CdSe quantum dot/Au NP systems [21–22]. Generally, we can state two major requirements for the protein adaptor approach binding NPs via the sulfur of the cysteine:

1. The NPs need to be able to covalently bind to thiols like Au or at least very strongly attach to thiols via physical forces.
2. The size of the NPs is limited to 4 nm on the lower end (Figure S3, Supporting Information File 1) and to 40 nm on the upper end [22].

If these requirements are fulfilled, Hcp1_cys3 can be applied to several systems as demonstrated for Au-, Fe_3_O_4_ and CoFe_2_O_4_ NPs and is therefore a variable molecule for the assembly of larger NP structures. However, larger hybrid structures on the micrometer scale can be obtained with recognizable order only when the NPs are prealigned by external magnetic forces as was shown for the Fe_3_O_4_ and CoFe_2_O_4_. The NPs in the hybrid structures show a preferable crystallographic orientation on the nanometer scale. The fiber formation can enhance the stability of the magnetic spin in the Fe_3_O_4_ NPs. In all other cases, Hcp1_cys3 acts as a glue and leads to self-assembly of the NPs into linear structures of limited length. This occurs until branches are formed by the statistical adsorption of two protein rings on one NP, forming a branch. Therefore, only short linear chains are feasible by this approach using understoichiometric Hcp1_cys3 concentrations and all larger structures contain branches. Nevertheless, the physical properties of these structures and the surface plasmon resonance still correspond to that of a linear structure and network structure. Therefore, assembly of NPs with defined interparticle distances using the toroidal cysteine-modified protein Hcp1_cys3 as a gluing unit is a promising approach towards different metallic NP chains and to explore their physical properties.

## Experimental

The Au NP, magnetite and the cobalt ferrite NPs were synthesized by the reported protocols of Slot [42] and Cabrera [36], respectively. All NPs systems were dispersible in water. The ligand exchange for CoFe_2_O_4_ NP was conducted by the modified protocol of Lees [41]. In the modified protocol, the precipitation with hexane/ethanol was replaced by centrifugation of the solution at 40000 rpm. The supernatant was discarded and precipitate was collected and redispersed in MeOH. The procedure was repeated three times. The solid was dried in a vacuum oven and could be easily dispersed in water. The storage buffer (50 mM Tris, 500 mM NaCl, 150 mM imidazol, 10% glycerol) of proteins (supplied by the Schreiber group) was removed with PD Spin Trap G-25 from GE Healthcare. The proteins were dissolved in water with 100 mM NaCl.

### Au NP Hcp1_cys3 network

The Au NPs were used directly after synthesis. The NP concentration was determined by measuring the absorbance of the plasmon peak at 520 nm by using the extinction coefficient of Au NPs with a diameter of 10.7 nm [43]. The Au network was prepared by adding the protein solution (the protein equivalent based on Au NP concentration) to the Au NP solution and incubating for 10 min, followed by fast mixing. The NaCl concentration in the Au NP mixture was adjusted to be 12 mM. For the kinetic investigation, UV–vis spectra were recorded at time intervals of 30 min over 24 h. The solution was directly employed for Raman spectroscopy and the catalytic reactions. For the catalytic reaction of 4-nitrophenol (NPA), a mixture of 3.3·10^−5^ M NPA, 166·10^−5^ M sodium borohydride was prepared. The mixture was placed in the UV–vis spectrometer. Au NPs or the Au network solution was added to the mixture with the end Au NP concentration of 1.8 nM. The solution was stirred during the entire measurement. The intensity at 400 nm was detected in time intervals of 5 s over 30 min.

### Fe_3_O_4_ Hcp1_cys3 network

An Fe_3_O_4_ NP solution with a concentration of 0.3 μM (based on the mass concentration of 0.5 mg/mL, diameter of 8 nm and density of magnetite bulk material of 5.2 g/cm^3^) was placed in a magnetic field of 0.5 T, which is parallel to the sample. The solution was incubated in the field for 1 h. Two equivalents Hcp1_cys3 based on the NP concentration were slowly added to the solution. The mixture was incubated overnight at room temperature. After 18 h, a carbon-covered TEM grid was added to the mixture to collect the species in the mixture for TEM and HRTEM. Finally, the solution with a TEM grid was frozen in liquid nitrogen for 10 min in the magnetic field. The frozen sample was removed and lyophilized under 0.01 mbar vacuum over 2 days. The reference sample was prepared following the same protocol without protein addition. A control sample was prepared by the same protocol as described for the Au Hcp1_cys3 network sample.

### CoFe_2_O_4_ Hcp1_cys3 network

A CoFe_2_O_4_ NP solution with a concentration of 2.9 μM (based on the mass concentration of 1.0 mg/mL, diameter of 5.5 nm and bulk density of of 5.3 g/cm^3^) was placed in a magnetic field of 0.5 T, which was parallel to the sample. The solution was incubated in the field for 1 h. Two equivalents Hcp1_cys3 based on NP the concentration were slowly added to the solution. The mixture was incubated overnight at room temperature. After 18 h, a carbon-covered TEM grid was added to the mixture to collect the species in the mixture for TEM and HRTEM. Finally, the solution with a TEM grid was frozen in liquid nitrogen for 10 min in the magnetic field. The frozen sample was removed and lyophilized under 0.01 mbar vacuum over 2 days.

### Methods

The absorption spectra were recorded on a UV–vis Cary 50 Probe spectrometer from Varian. The protein concentration was calculated by measuring the absorbance at 280 nm with a NanoDrop^®^ND-1000 from PEQLAB and using an extinction coefficient of 24,200 M^−1^cm^−1^. TEM and cryo-TEM images were collected using a Zeiss Libra120 TEM operated at 120 keV and a Zeiss EM922 Omega operating at 200 keV. For Au NP samples, a volume of 10 µL was transferred onto a glow-discharged carbon-coated copper grid. After 10 min, the drop was removed by filter paper. The Raman spectra were collected with a Perkin-Elmer Raman Station 100 by measuring the Au network in a quartz cuvette with a resolution of 2 cm^−1^. For the magnetite samples, a high frequency setup of magnetic field of ±0.5 T was employed. The SEM images and EDX analyses were performed utilizing a Hitachi TM 3000 microscope. HRTEM images were collected using a JEOL JEM-2200FS microscope at 200 keV. The analysis of the HRTEM images were realized by means of the Digital Micrograph (Gatan, USA) and JEMS (version: 3.5930U2010) software. Visualization of the magnetite crystal models was performed with the VESTA 3 software. The magnetic measurement was accomplished with a SQUID magnetometer, type MPMS XL5 from Quantum Design. The magnetization (*M*) was recorded at 300 K and 2 K between 50000 and −50000 Oe. Zero-field-cooled (ZFC) measurements were carried out by cooling the sample from room temperature to 2 K in zero magnetic field, then a static magnetic field of 10 mT was applied. *M*_ZFC_ was measured during warming up from 2 to 300 K. The field-cooled measurement was carried out by applying a magnetic field of 10 mT, and the *M*_FC_ was recorded during the subsequent cooling to 2 K.

## Supporting Information

File 1 Nanoparticle references and interparticle distance.
