# Supplementary material for: Hemolysin coregulated protein 1 as a molecular gluing unit for the assembly of nanoparticle hybrid structures
Source: Beilstein J Nanotechnol. 2016 Mar 4;7:351–63. doi: 10.3762/bjnano.7.32 (PMC4901536; doi:10.3762/bjnano.7.32)
Supplement: File 1 — Nanoparticle references and interparticle distance. [file Beilstein_J_Nanotechnol-07-351-s001.pdf]

Supporting Information

for

Hemolysin coregulated protein 1 as a molecular gluing unit for the assembly of nanoparticle hybrid structures

Tuan Anh Pham[1], Andreas Schreiber[2], Elena V. Sturm (née Rosseeva) [1], Stefan Schiller*[2], and Helmut Cölfen[*1]

Address: [1]Department of Chemistry, Physical Chemistry, University of Konstanz, Universitätstrasse 10, D-78457 Konstanz, Germany, and [2]Zentrum für Biosystemanalyse (ZBSA), Albert-Ludwigs-Universität Freiburg, Habsburgerstrasse 49, D-79104 Freiburg, Germany

Email: Helmut Cölfen* - helmut.coelfen@uni-konstanz.de; Stefan Schiller* - Stefan.Schiller@FRIAS.Uni-Freiburg.de

* Corresponding author

Nanoparticle references and interparticle distance

1. Gold nanoparticle (Au NP)

The synthesized Au NPs were investigated in TEM, as shown in Figure S1.

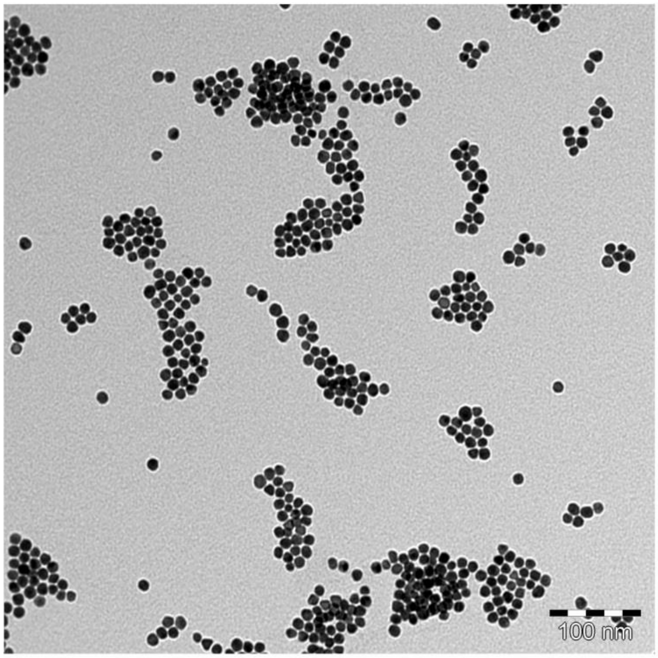

Figure S1: TEM image of Au NPs with a mean size of 10.7 ± 2.0 nm.

2. Interparticle distance of Au NPs

The interparticle distance of Au NPs in the TEM image of Figure S2 was measured using ImageJ software. An average value of 2.8 ± 0.6 nm could be determined.

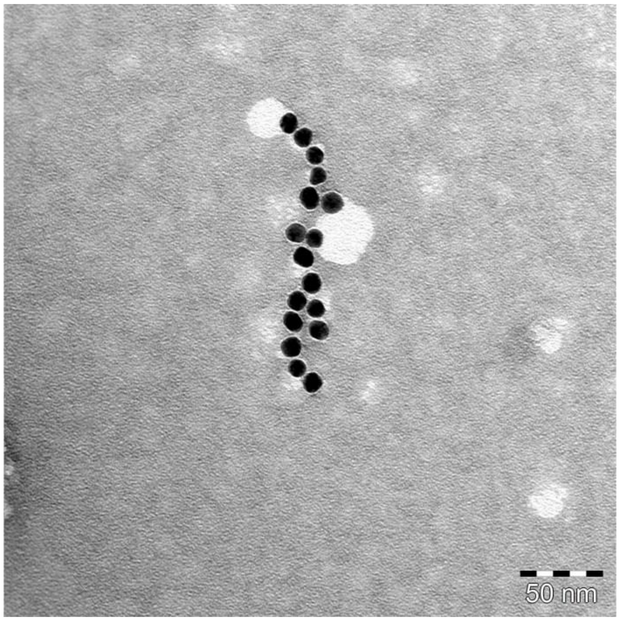

Figure S2: TEM image of Au NPs chain from Au Hcp1_cys3 network sample.

3. Calculation of interparticle distance

For the calculation of interparticle distance we assume a perfect sphere with a diameter of the size of the NP. The maximal penetration depth of the sphere into the rigid ring of the protein cavity can be calculated by the trigonometric function in Equation S1 below:

$$a = \sqrt{c^2 - b^2} \qquad (1)$$

with c = 5.35 nm; radius of our Au NP and b = 2 nm, radius of the Hcp1_cys3 cavity a penetration depth (a) of 0.39 nm is calculated. For Fe_3O_4 NP with c = 4 nm and $CoFe_2O_4$ NP with c = 2.75 nm an a value of 0.54 nm and 0.86 nm can be obtained. With this value and the height of Hcp1 with 4.4 nm an interparticle distance between two NPs of 3.62 nm for Au NP, 3.32 nm for Fe_3O_4 NP and 2.67 nm for $CoFe_2O_4$ NP can be calculated.

For Au NP with 4.0 nm in diameter *a* value of 2 nm can be obtained. Two NPs of this size can fit perfectly into the cavity of the Hcp1_cys3 structure and the resulting interparticle decreases to 0.4 nm, as shown in Figure S3.

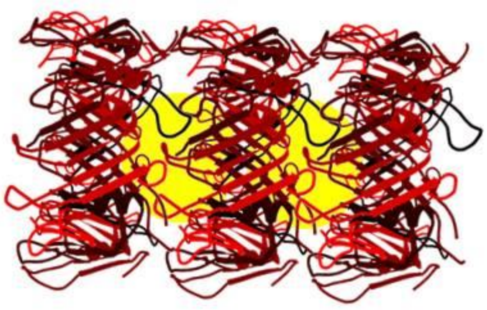

Figure S3: Two nanoparticles with a diameter of 4 nm can fit into the Hcp1_cys3 protein cavity.

4. Magnetite nanoparticle (Fe_3O_4 NP)

The size of magnetite nanoparticles (Fe_3O_4 NPs) was determined in the TEM image of Figure S4.

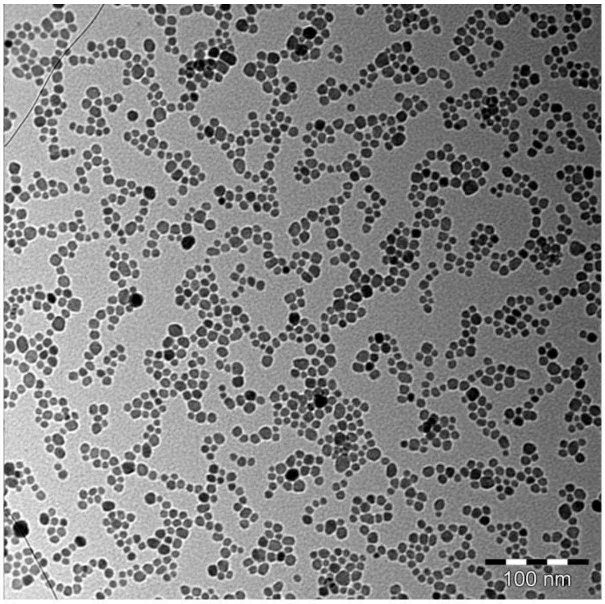

Figure S4: TEM image of Fe_3O_4 NPs with a size of about 8 nm.

5. Fe_3O_4 Hcp1_cys3 sample without alignment in a magnetic field

A Fe_3O_4 Hcp1_cys3 sample was prepared by the same protocol for Au Hcp1_cys3 network sample without the utilization of an external magnetic field. The TEM result is shown in Figure S5.

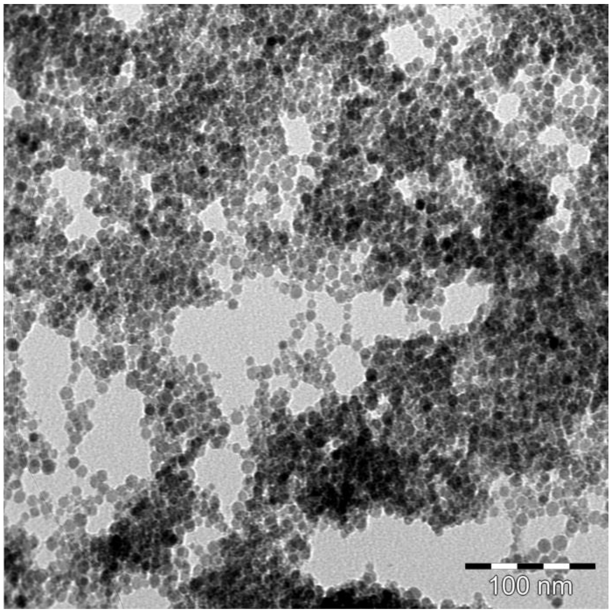

Figure S5: TEM image of a Fe_3O_4 Hcp1_cys3 sample prepared by the mixing protocol for Au_Hcp1_cys3 network sample.

6. Interparticle distance of Fe_3O_4 NPs

The interparticle distance of Fe_3O_4 NPs in the Fe_3O_4 Hcp1_cys3 fiber-like structure after the lyophilization was measured in the HRTEM image of Figure S7 using Digital Micrograph™ software. An average value of 1.5 ± 0.5 nm could be determined.

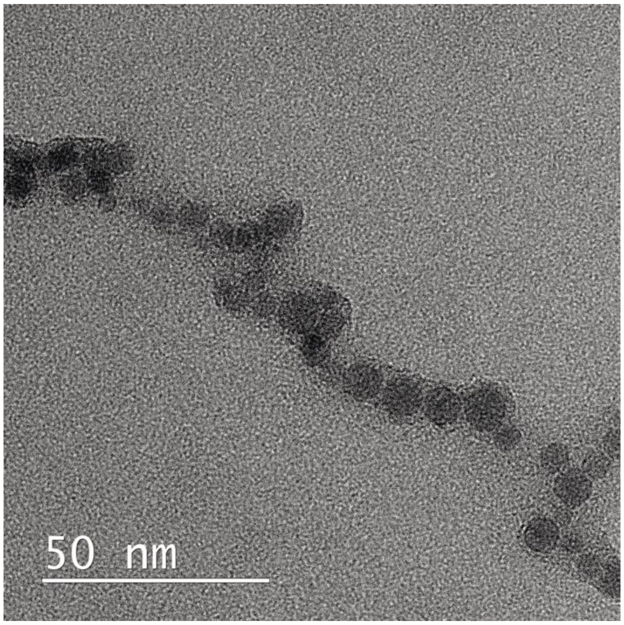

Figure S6: HRTEM image of Fe_3O_4 NPs in the Fe_3O_4 Hcp1_cys3 fiber-like structure.

7. Cobalt ferrrite nanoparticle ($CoFe_2O_4$ NP)

The cobalt nanoparticles ($CoFe_2O_4$ NPs) show in the TEM image of Figure S7 a size of about 5.5 nm.

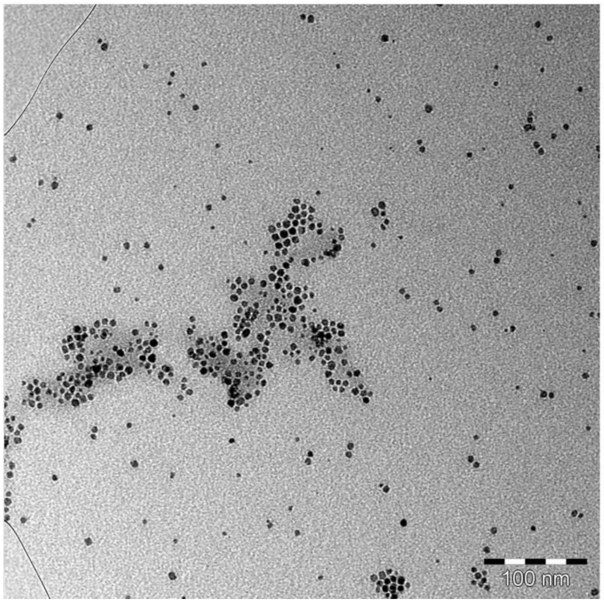

Figure S7: TEM image of $CoFe_2O_4$ NPs.

8. Interparticle distance of $CoFe_2O_4$ NPs

The interparticle distance of $CoFe_2O_4$ NPs in the $CoFe_2O_4$ Hcp1_cys3 fiber-like structure after the lyophilization sample was measured in the HRTEM image of Figure S8 using Digital Micrograph™ software. An average value of 0.8 ± 0.3 nm could be determined for the interparticle distance of $CoFe_2O_4$ in the $CoFe_2O_4$ Hcp1_cys3 fiber.

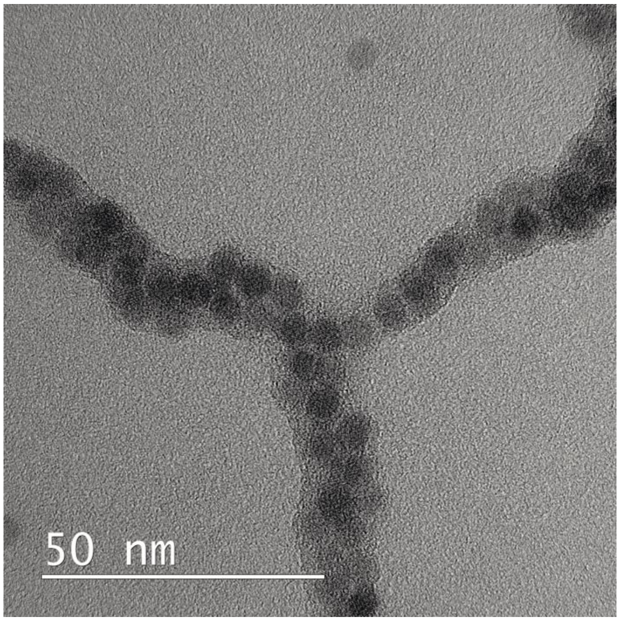

Figure S8: HRTEM image of $CoFe_2O_4$ NPs in the $CoFe_2O_4$ Hcp1_cys3 fiber.

9. Au Hcp1_cys3 sample with under-stoichiometric protein concentration

The TEM image of Au Hcp1_cys3 network sample containing 0.6 equivalent Hcp1_cys3 protein and 12 mM NaCl concentration in Figure S9 shows short linear chains with 2–10 Au NPs and also free Au NPs.

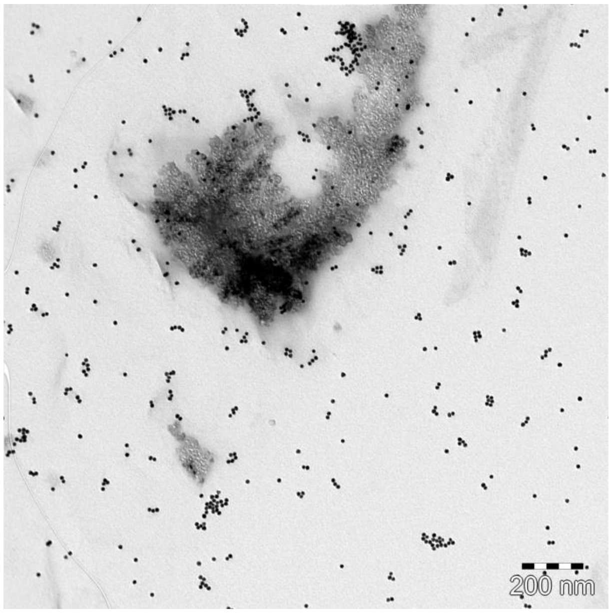

Figure S9: TEM image of Au Hcp1_cys3 network sample containing 0.6 equivalent Hcp1_cys3 protein and 12 mM NaCl concentration.

10. Fe_3O_4 NPs in the Fe_3O_4 Hcp1_cys3 fiber-like structure

The HRTEM image of Fe_3O_4 Hcp1-cys3 fiber in Figure S10 shows an organic layer around the Fe_3O_4 NPs indicated by red dashed line. A layer thickness (distance between NP and the line) of 4–4.5 nm is determined, which corresponds to the height of the Hcp1_cys3 protein. This result confirms, as already concluded from the Au Hcp1_cys3 network structure, a statistical adsorption of more than one Hcp1_cys3 protein on one NP, which leads to Hcp1_cys3 location in between two NPs and on the sides of the Fe_3O_4 NPs. This adsorption triggers the branch formation of Au NPs chains.

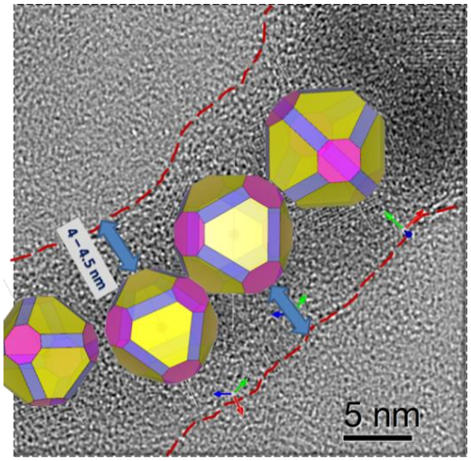

Figure S10: HRTEM image of a Fe_3O_4 NPs chain in the Fe_3O_4 Hcp1_cys3 fiber.
